# 2-(2-Nitro­phenyl­sulfin­yl)acetonitrile

**DOI:** 10.1107/S1600536813004832

**Published:** 2013-02-23

**Authors:** Sabrina Benmebarek, Mhamed Boudraa, Sofiane Bouacida, Hocine Merazig

**Affiliations:** a Unité de Recherche de Chimie de l’Environnement et Moléculaire Structurale (CHEMS), Université Mentouri–Constantine, 25000 , Algeria; b Département Sciences de la Matière, Faculté des Sciences Exactes et Sciences de la Nature et de la Vie, Université Oum El Bouaghi, Algeria

## Abstract

In the title compound, C_8_H_6_N_2_O_3_S, the dihedral angle between the nitro group and the benzene ring is 6.76 (9)°. The bond-angle sum at the S atom is 308.1°. In the crystal, mol­ecules are linked by C—H⋯O hydrogen bonds to generate (010) sheets. The crystal studied was found to be a racemic twin.

## Related literature

For a related structure and background to sulfoxides, see: Benmebarek *et al.* (2012 ▶). For related structures see: Yan (2010 ▶); Kobayashi *et al.* (2003 ▶).
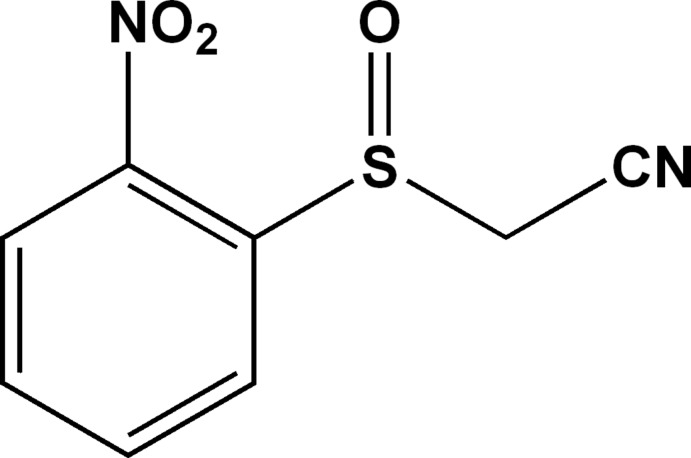


## Experimental

### 

#### Crystal data

C_8_H_6_N_2_O_3_S
*M* *_r_* = 210.21
Orthorhombic, 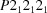

*a* = 5.4114 (2) Å
*b* = 10.7602 (4) Å
*c* = 15.1837 (5) Å
*V* = 884.11 (5) Å^3^
*Z* = 4
Mo *K*α radiation
μ = 0.35 mm^−1^
*T* = 295 K
0.26 × 0.2 × 0.15 mm

#### Data collection

Bruker APEXII CCD diffractometer
8401 measured reflections
2348 independent reflections
2222 reflections with *I* > 2σ(*I*)
*R*_int_ = 0.016

#### Refinement

*R*[*F* ^2^ > 2σ(*F* ^2^)] = 0.027
*wR*(*F* ^2^) = 0.070
*S* = 1.05
2348 reflections
128 parameters
H-atom parameters constrained
Δρ_max_ = 0.21 e Å^−3^
Δρ_min_ = −0.18 e Å^−3^
Absolute structure: Flack (1983 ▶), 1379 Friedel pairs
Flack parameter: 0.53 (1)

### 

Data collection: *APEX2* (Bruker, 2011 ▶); cell refinement: *APEX2*; data reduction: *APEX2*; program(s) used to solve structure: *SIR2002* (Burla *et al.*, 2005 ▶); program(s) used to refine structure: *SHELXL97* (Sheldrick, 2008 ▶); molecular graphics: *ORTEP-3* (Farrugia, 2012 ▶) and *DIAMOND* (Brandenburg & Berndt, 2001 ▶); software used to prepare material for publication: *WinGX* (Farrugia, 2012 ▶).

## Supplementary Material

Crystal structure: contains datablock(s) global, I. DOI: 10.1107/S1600536813004832/hb7044sup1.cif

Structure factors: contains datablock(s) I. DOI: 10.1107/S1600536813004832/hb7044Isup2.hkl

Supplementary material file. DOI: 10.1107/S1600536813004832/hb7044Isup3.cml

Additional supplementary materials:  crystallographic information; 3D view; checkCIF report

## Figures and Tables

**Table 1:** Hydrogen-bond geometry (Å, °)

*D*—H⋯*A*	*D*—H	H⋯*A*	*D*⋯*A*	*D*—H⋯*A*
C6—H6⋯O2^i^	0.93	2.41	3.3198 (18)	165
C7—H7*A*⋯O2^ii^	0.97	2.50	3.1190 (19)	122

Symmetry codes: (i) 
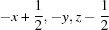
; (ii) 
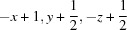
.

**Fig. 1.**
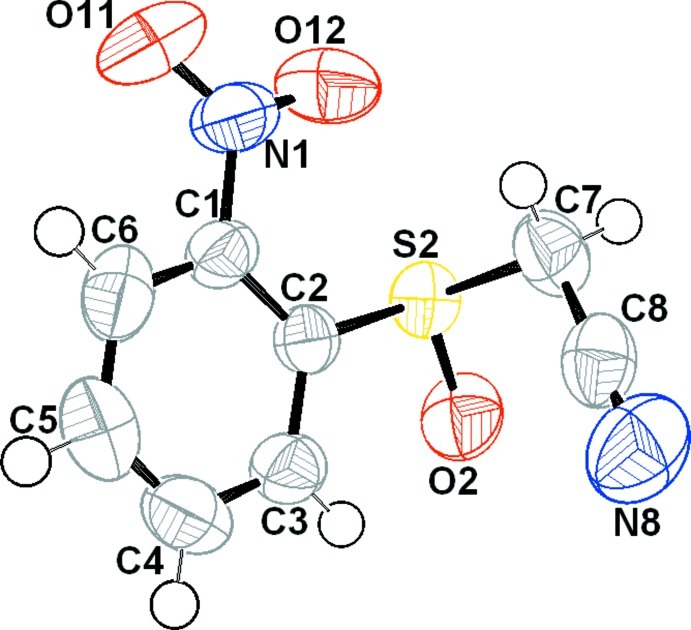
View of the asymetric unit of, (I), with displacement ellipsoids drawn at the 50% probability level.

**Fig. 2.**
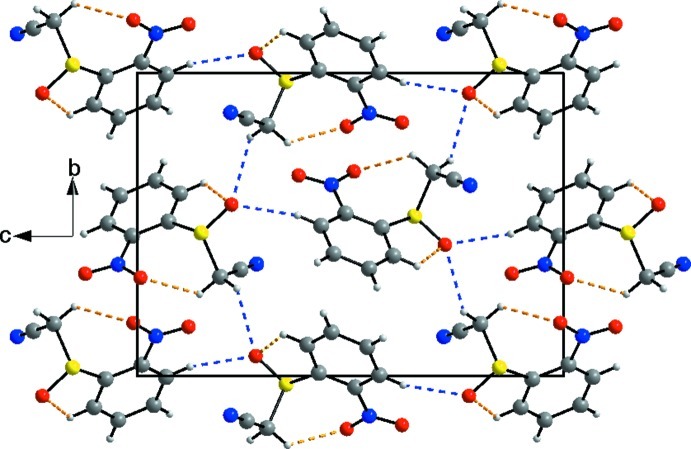
Packing diagram of (I) viwed down [100] showing the hydrogen bonding in alterning layers parallel to (010) planes.

## supplementary crystallographic information

### Comment

As part of our ongoing studies on the synthesis, structures and biological activity of organometallic sulfanilamide complexes (Benmebarek *et al.* 2012), we have synthesized and determined the crystal structure of the title compound, (I). The molecular geometry and the atom-numbering scheme are shown in Fig 1. The bond angle sum at the S atom is 308.1°. The nitro group forms a dihedral angle of 6.76 (9)° with the benzene ring, which is very different to that found in 2-(methylsulfinyl)benzamide (25.6°) (Yan, 2010) and in benzamide (26.3°) (Kobayashi *et al.*, 2003), and similar to chloromethylsulfinyl-2-nitrobenzene (2.7°) (Benmebarek *et al.*, 2012). The crystal structure features C—H···O hydrogen bonds (Fig. 2) forming (010) sheets.

### Experimental

Chloromethylsulfinyl-2-nitrobenzene (2.196 g, 10 mmol) obtained according to established procedures (Benmebarek *et al.*, 2012) and potassium cyanide (0.65 g, 10 mmol) were dissolved in 75 ml aqua ethanol solution (25 ml water + 50 ml ethanol) and refluxed for 3 h under continuous stirring. Then the obtained product was evaporated at room temperature to dryness. The residue was diluted in 50 ml pure ethanol. After a few days, colourless blocks were recovered, as the solvent slowly evaporated.

### Refinement

All non-H atoms were refined with anisotropic atomic displacement parameters. Approximate positions for all H atoms were first obtained from the difference electron density map. However, the H atoms were situated into idealized positions and the H-atoms have been refined within the riding atom approximation. The applied constraints were as follow: C_aryl_—H_aryl_ = 0.93 Å and C_methylene_—H_methylene_ = 0.97 Å. *U*_iso_(H_aryl_/_methylene_) = 1.2*U*_eq_(C_aryl_/C_methylene_).

### Crystal data

**Table:**

C_8_H_6_N_2_O_3_S	*F*(000) = 432
*M**_r_* = 210.21	*D*_x_ = 1.579 Mg m^−^^3^
Orthorhombic, *P*2_1_2_1_2_1_	Mo *K*α radiation, λ = 0.71073 Å
Hall symbol: P 2ac 2ab	Cell parameters from 6191 reflections
*a* = 5.4114 (2) Å	θ = 2.7–29.1°
*b* = 10.7602 (4) Å	µ = 0.35 mm^−^^1^
*c* = 15.1837 (5) Å	*T* = 295 K
*V* = 884.11 (5) Å^3^	Block, colourless
*Z* = 4	0.26 × 0.2 × 0.15 mm

### Data collection

**Table:**

Bruker APEXII CCD diffractometer	2222 reflections with *I* > 2σ(*I*)
Radiation source: sealed tube	*R*_int_ = 0.016
Graphite monochromator	θ_max_ = 29.1°, θ_min_ = 4.0°
φ and ω scans	*h* = −6→7
8401 measured reflections	*k* = −14→14
2348 independent reflections	*l* = −20→20

### Refinement

**Table:**

Refinement on *F*^2^	Secondary atom site location: difference Fourier map
Least-squares matrix: full	Hydrogen site location: inferred from neighbouring sites
*R*[*F*^2^ > 2σ(*F*^2^)] = 0.027	H-atom parameters constrained
*wR*(*F*^2^) = 0.070	*w* = 1/[σ^2^(*F*_o_^2^) + (0.0327*P*)^2^ + 0.2515*P*] where *P* = (*F*_o_^2^ + 2*F*_c_^2^)/3
*S* = 1.05	(Δ/σ)_max_ = 0.001
2348 reflections	Δρ_max_ = 0.21 e Å^−^^3^
128 parameters	Δρ_min_ = −0.18 e Å^−^^3^
0 restraints	Absolute structure: Flack (1983), 1379 Friedel pairs
Primary atom site location: structure-invariant direct methods	Flack parameter: 0.53 (1)

### Special details

**Table:**

Geometry. All e.s.d.'s (except the e.s.d. in the dihedral angle between two l.s. planes) are estimated using the full covariance matrix. The cell e.s.d.'s are taken into account individually in the estimation of e.s.d.'s in distances, angles and torsion angles; correlations between e.s.d.'s in cell parameters are only used when they are defined by crystal symmetry. An approximate (isotropic) treatment of cell e.s.d.'s is used for estimating e.s.d.'s involving l.s. planes.
Refinement. Refinement of *F*^2^ against ALL reflections. The weighted *R*-factor *wR* and goodness of fit *S* are based on *F*^2^, conventional *R*-factors *R* are based on *F*, with *F* set to zero for negative *F*^2^. The threshold expression of *F*^2^ > σ(*F*^2^) is used only for calculating *R*-factors(gt) *etc*. and is not relevant to the choice of reflections for refinement. *R*-factors based on *F*^2^ are statistically about twice as large as those based on *F*, and *R*- factors based on ALL data will be even larger.

### Fractional atomic coordinates and isotropic or equivalent isotropic displacement parameters (Å^2^)

**Table:**

	*x*	*y*	*z*	*U*_iso_*/*U*_eq_	
S2	0.53769 (6)	0.02767 (3)	0.15053 (2)	0.02770 (9)	
O2	0.5137 (2)	−0.06613 (11)	0.22173 (7)	0.0395 (3)	
O11	0.4219 (4)	0.16230 (15)	−0.12118 (8)	0.0678 (5)	
O12	0.5834 (2)	0.17541 (11)	0.00821 (9)	0.0453 (3)	
N1	0.4351 (3)	0.13166 (12)	−0.04412 (9)	0.0352 (3)	
N8	−0.0001 (3)	0.12996 (17)	0.28063 (10)	0.0515 (4)	
C1	0.2643 (3)	0.03718 (13)	−0.01153 (9)	0.0263 (3)	
C2	0.2906 (2)	−0.01016 (12)	0.07377 (8)	0.0235 (2)	
C3	0.1293 (3)	−0.10289 (13)	0.10054 (10)	0.0294 (3)	
H3	0.1443	−0.137	0.1566	0.035*	
C4	−0.0546 (3)	−0.14513 (14)	0.04407 (11)	0.0357 (3)	
H4	−0.1622	−0.2071	0.0628	0.043*	
C5	−0.0795 (3)	−0.09610 (15)	−0.03950 (11)	0.0376 (3)	
H5	−0.2034	−0.125	−0.0767	0.045*	
C6	0.0809 (3)	−0.00355 (14)	−0.06796 (9)	0.0343 (3)	
H6	0.0651	0.0304	−0.124	0.041*	
C7	0.4040 (3)	0.17220 (14)	0.19493 (11)	0.0341 (3)	
H7A	0.5225	0.2113	0.234	0.041*	
H7B	0.3711	0.2292	0.1468	0.041*	
C8	0.1763 (3)	0.14879 (15)	0.24274 (10)	0.0330 (3)	

### Atomic displacement parameters (Å^2^)

**Table:**

	*U*^11^	*U*^22^	*U*^33^	*U*^12^	*U*^13^	*U*^23^
S2	0.02549 (15)	0.02869 (16)	0.02891 (16)	0.00442 (13)	−0.00081 (13)	−0.00137 (13)
C1	0.0308 (6)	0.0236 (6)	0.0246 (6)	0.0010 (5)	0.0058 (5)	0.0006 (5)
O2	0.0509 (7)	0.0360 (5)	0.0316 (5)	0.0073 (5)	−0.0070 (5)	0.0061 (4)
C3	0.0344 (7)	0.0242 (6)	0.0296 (7)	−0.0001 (5)	0.0047 (6)	0.0007 (5)
O12	0.0399 (7)	0.0423 (6)	0.0537 (7)	−0.0116 (5)	0.0041 (6)	0.0087 (5)
C5	0.0359 (8)	0.0373 (8)	0.0395 (8)	0.0008 (7)	−0.0071 (7)	−0.0108 (6)
N1	0.0417 (7)	0.0303 (6)	0.0336 (6)	−0.0008 (6)	0.0104 (6)	0.0055 (5)
N8	0.0469 (10)	0.0585 (9)	0.0492 (8)	0.0082 (8)	0.0136 (8)	−0.0025 (7)
C2	0.0249 (6)	0.0216 (6)	0.0241 (6)	0.0019 (5)	0.0024 (5)	−0.0019 (5)
C4	0.0326 (7)	0.0291 (6)	0.0453 (8)	−0.0061 (6)	0.0045 (7)	−0.0038 (6)
C6	0.0440 (8)	0.0347 (7)	0.0244 (6)	0.0065 (6)	−0.0034 (6)	−0.0033 (5)
O11	0.0987 (13)	0.0692 (10)	0.0354 (6)	−0.0252 (9)	0.0125 (8)	0.0169 (6)
C8	0.0360 (8)	0.0329 (7)	0.0299 (7)	0.0085 (6)	−0.0016 (6)	−0.0045 (6)
C7	0.0337 (8)	0.0286 (7)	0.0401 (8)	0.0006 (6)	0.0013 (6)	−0.0080 (6)

### Geometric parameters (Å, º)

**Table:**

S2—O2	1.4846 (11)	C5—C4	1.381 (2)
S2—C2	1.8197 (13)	C5—C6	1.390 (2)
S2—C7	1.8429 (15)	C5—H5	0.93
C1—C6	1.382 (2)	N1—O11	1.2177 (17)
C1—C2	1.3989 (18)	N8—C8	1.133 (2)
C1—N1	1.4606 (18)	C4—H4	0.93
C3—C2	1.3867 (19)	C6—H6	0.93
C3—C4	1.390 (2)	C8—C7	1.452 (2)
C3—H3	0.93	C7—H7A	0.97
O12—N1	1.2235 (19)	C7—H7B	0.97
			
O2—S2—C2	104.49 (6)	C3—C2—S2	115.83 (10)
O2—S2—C7	105.84 (7)	C1—C2—S2	125.91 (10)
C2—S2—C7	97.74 (7)	C5—C4—C3	120.78 (15)
C6—C1—C2	122.12 (13)	C5—C4—H4	119.6
C6—C1—N1	117.72 (12)	C3—C4—H4	119.6
C2—C1—N1	120.16 (12)	C1—C6—C5	118.86 (13)
C2—C3—C4	120.35 (13)	C1—C6—H6	120.6
C2—C3—H3	119.8	C5—C6—H6	120.6
C4—C3—H3	119.8	N8—C8—C7	179.36 (19)
C4—C5—C6	119.90 (15)	C8—C7—S2	111.69 (11)
C4—C5—H5	120	C8—C7—H7A	109.3
C6—C5—H5	120	S2—C7—H7A	109.3
O11—N1—O12	123.94 (15)	C8—C7—H7B	109.3
O11—N1—C1	118.48 (15)	S2—C7—H7B	109.3
O12—N1—C1	117.58 (12)	H7A—C7—H7B	107.9
C3—C2—C1	117.97 (13)		
			
C6—C1—N1—O11	−6.2 (2)	C7—S2—C2—C3	−102.58 (11)
C2—C1—N1—O11	173.11 (15)	O2—S2—C2—C1	−167.63 (11)
C6—C1—N1—O12	173.76 (14)	C7—S2—C2—C1	83.73 (12)
C2—C1—N1—O12	−7.0 (2)	C6—C5—C4—C3	0.1 (2)
C4—C3—C2—C1	−1.0 (2)	C2—C3—C4—C5	0.2 (2)
C4—C3—C2—S2	−175.24 (11)	C2—C1—C6—C5	−1.2 (2)
C6—C1—C2—C3	1.5 (2)	N1—C1—C6—C5	178.08 (13)
N1—C1—C2—C3	−177.73 (12)	C4—C5—C6—C1	0.3 (2)
C6—C1—C2—S2	175.08 (11)	O2—S2—C7—C8	−42.69 (13)
N1—C1—C2—S2	−4.16 (18)	C2—S2—C7—C8	64.82 (12)
O2—S2—C2—C3	6.06 (12)		

### Hydrogen-bond geometry (Å, º)

**Table:**

*D*—H···*A*	*D*—H	H···*A*	*D*···*A*	*D*—H···*A*
C6—H6···O2^i^	0.93	2.41	3.3198 (18)	165
C7—H7*A*···O2^ii^	0.97	2.50	3.1190 (19)	122

Symmetry codes: (i) −*x*+1/2, −*y*, *z*−1/2; (ii) −*x*+1, *y*+1/2, −*z*+1/2.
